# Adverse childhood experiences as risk factors for recurrent admissions in young psychiatric inpatients

**DOI:** 10.3389/fpsyt.2022.988695

**Published:** 2022-11-29

**Authors:** Matthäus Fellinger, Philipp Knasmüller, Krisztina Kocsis-Bogar, Andreas Wippel, Laura Fragner, Dunja Mairhofer, Paulus Hochgatterer, Martin Aigner

**Affiliations:** ^1^Clinical Division of Social Psychiatry, Department of Psychiatry and Psychotherapy, Medical University of Vienna, Vienna, Austria; ^2^Department of Psychiatry and Medical Psychotherapy, University Hospital Tulln, Tulln, Austria; ^3^Department of General Psychiatry, Clinic Landstraße, Vienna Healthcare Group, Vienna, Austria; ^4^Department of Child and Adolescent Psychiatry, Medical University of Vienna, Vienna, Austria; ^5^Department of Child and Adolescent Psychiatry, University Hospital Tulln, Tulln, Austria; ^6^Karl Landsteiner Private University of Health Sciences, Krems an der Donau, Austria

**Keywords:** risk, adverse childhood experiences, childhood trauma, child psychiatry, transition to adult care, adolescent psychiatry, mental health services

## Abstract

**Background:**

Patients who require psychiatric inpatient treatment early in life are a particularly at-risk population. Factors such as adverse childhood experiences (ACEs) are, however, not well studied in those requiring psychiatric inpatient treatment during both childhood or adolescence and adulthood. Thus, the aim of the current study was to investigate, in young adult inpatients, the risk factors for prior admissions in Child and Adolescent Psychiatry, with a focus on ACEs.

**Materials and methods:**

An explorative population-based systematic chart investigation of psychiatric inpatients aged 18–25 was conducted at the University Hospital Tulln, Austria. Data analysis was done with descriptive methods and Pearson’s chi- squared-, Fisher’s exact-, Mann–Whitney-*U*-tests and predictive logistic regression models.

**Results:**

The sample comprised 390 inpatients (51.8% female), with an average age of 20 years at first psychiatric hospital admission. Those with a former child and adolescent psychiatry inpatient treatment (10.3%) were predominantly female (77.5%). Their number of documented ACEs was increased compared to those without former child and adolescent psychiatry admissions (2 vs 1.1), with up to twice as many experiences of family dysfunction, neglect or abuse. Sexual abuse (OR: 3.0), having been an adopted or fostered child (OR: 4.5), and female sex (OR: 3.0) were identified as main risk factors. Furthermore, former child and adolescent psychiatry inpatients suffered from higher rates of psychosomatic or personality disorders, comorbidities and functional impairment, and were readmitted twice as often in young adulthood.

**Conclusion:**

Young adult inpatients with reoccurring psychiatric inpatient treatments have increased rates of severe ACEs. Thus, special attention should be given to identifying ACEs, evaluating needs for psychosocial support and therapy, and meeting these needs after discharge.

## Introduction

Patients who require psychiatric inpatient treatment (PIT) early in life seem to be a particularly vulnerable group with increased risks of suicide, chronic psychiatric disorders, comorbidity and ongoing service use (1–6). Psychiatric inpatient treatment, in general, is regarded as an intensive intervention indicating a severe disability due to either a serious mental disorder or health-threatening psychosocial or trauma-related factors (7–9). In comparison to other types of care it is more restrictive and is usually applied when other types of treatment have been unsuccessful, or the safety of oneself or others is at risk (10, 11). Furthermore, it is by far the most expensive form of care, consuming up to 90% of the mental health budget (e.g., US $3.5 billion annual cost), even for patients of a young age (3–20 years) (12, 13).

There is a wealth of literature investigating risk factors for mental disorders, and in recent years adverse childhood experiences (ACEs) have been increasingly acknowledged to play an important role (14–19). However, there are few studies examining risk factors for psychiatric inpatient treatment and its reoccurrence at a young age, and even fewer that focus on ACEs. ACEs are defined as negative early experiences in the psychosocial environment, including the main categories of childhood abuse, neglect and family dysfunction such as parental divorce, foster care or death of a parent (14). Its experience, particularly when being present over a longer period of time and in high amounts, has been shown to cause an excessive activation of the stress response system (20). This so-called “toxic stress” has been shown to result in multiple negative effects on a child’s neuropsychological development and metabolic, immune, and cardiovascular systems and is a relevant explanation for the association of ACEs and poor health outcomes (21). Generally, the association between ACEs and mental illness as well as self-directed violence appears to be stronger than that between ACEs and adverse somatic health consequences, including obesity, cancer or heart disease (15). When describing influencing factors, it should be mentioned that biological sex plays an important role in experiencing childhood trauma and adverse life events as well as its trajectories. Girls are more likely to be exposed to sexual, whereas boys to physical abuse (22, 23). There is evidence that girls tend to be exposed to a broader range of adversities and consequently be at a higher risk for mental illness. Additionally, there is a greater tendency for females with ACEs to show internalizing and for males with ACEs to show externalizing behaviors (22). ACEs are associated with depressive symptoms only in adolescent girls, but not in boys (24), whereas with self-reported delinquency in boys, but not girls (25). When being distressed it is known that women in both adolescence (1) and adulthood (26) are more likely to contact inpatient services.

Thus, due to the negative impact of ACEs on mental health and the less stable and supportive environment in which those affected from ACE often find themselves (27–29) it is reasonable to assume that those, who require psychiatric treatment early in life are more likely to be affected by ACEs and being female. Accordingly, elevated rates of ACEs and worse outcomes were found in those with Child and Adolescent Psychiatry (CAP) admissions. One study found that 85% of those with previous admissions in child and adolescent psychiatry reported at least one adverse life event and were shown to have experienced ACEs (2.2 vs 0.6) nearly four times as often as healthy adolescents (7). A retrospective chart review has shown that CAP inpatients who experience abuse have a higher rate of psychiatric comorbidity and longer admissions, however, without examining childhood family adversities, except for growing up with an adoptive parent or in custody (30). The only cohort study that has previously assessed childhood predictors of psychiatric inpatient treatment in adolescence and young adulthood is a Finnish 1981 birth cohort study that followed up over 5,000; 8-year-old children; 6.2% of males and 4.1% of females were subsequently admitted as psychiatric inpatients between the ages of 13 and 24. Psychiatric admission was strongly predicted by self-reported depressive symptoms in female 8-year-old, a non-intact family situation in male 8-year-old, and emotional and conduct problems in both male and female children. Growing up in a non-intact family predicted the number of hospital treatment in both sexes, but further family adversities were not examined (8).

One of the first studies assessing long term outcomes for former CAP inpatients reported that former male CAP inpatients suffered from a higher rate of severe delinquency, mental disorders and maladjustment compared to the general population (31). In more recent studies, however, only CAP inpatients with comorbid depression and conduct disorder were found to have an increased use of psychiatric inpatient and criminal justice services, or heightened health care costs in adulthood (3). The estimated rate of CAP inpatients with subsequent psychiatric readmissions in adulthood varies between 20 and 40% (6, 32, 33). Based on results from a large European study, readmission rates appear particularly increased when patients are diagnosed with a schizophrenia spectrum (fourfold), personality (two-and-a-half-fold) or developmental (twofold) disorder (34). Compared to the general population, former CAP inpatients have a fourfold increased need for psychiatric inpatient treatment between the ages of 18 and 31, and 13 years after discharge they are twice as likely to suffer from depression and four times as likely to be diagnosed with a personality disorder (6).

While there are a number of studies investigating children and adolescents with psychiatric inpatient treatment, there is still minimal research addressing young adults. This is despite the fact that young adulthood is acknowledged to be an important developmental period, with significant psychosocial and neurobiological changes crucial for a healthy transition into adulthood (35). Partly due to changes in our society such as prolonged periods of education, as well as later ages at which one has stable relationships or raises children it is an age with intensive psychodynamic processes that are influenced by formative life experiences and changes such as entering into a (permanent) partnership, learning to live independently, or starting a career (35). Despite the relevance for treatment and care planning, there is a lack of data on risk factors for early and recurring hospital admissions in the group of young adults. Although there is good evidence of ACEs as a risk factor for mental health, the corresponding impact on (former) treatment use in this age cohort has not yet been studied. Furthermore, previous studies in other age groups relied mainly on cumulative ACE scores (14–16, 18, 24, 25), without examining the contribution of the specific ACEs to mental health problems or hospitalization. Therefore, this study aimed to address this gap in the literature and to compare predictors of childhood hospitalization in young adult inpatients. In particular, the study focused on ACE in terms of a vulnerability-risk model, assuming that ACEs are significant risk factors for psychiatric hospitalization in childhood, adolescence and adulthood. Based on the studies cited above, we hypothesized that (1) increased rates of all specific (abuse, neglect, and family dysfunction) and cumulative ACEs (2) female sex (3) schizophrenia spectrum, personality and developmental disorders and (4) a lower global functioning would be more present in those young adult psychiatric inpatients with former childhood and adolescent inpatient care than those without former CAP care.

## Materials and methods

### Study design and participants

The study was an explorative retrospective investigation that took place at the Department of Adult Psychiatry of the University Hospital Tulln in Lower Austria. The location was deemed appropriate for this investigation as the department shares treatment responsibility for an overlapping catchment area with the Department of Child and Adolescent Psychiatry at the University Hospital Tulln. All patients that had received at least 1 day of PIT at the Department of Adult Psychiatry of the University Hospital Tulln between 2010 and 2015, and who were aged 18–25 (born between 1990 and 1997), were assessed as part of the study.

In general, all patients who are admitted at the Department of Adult Psychiatry of the University Hospital Tulln are assessed by a psychiatrist upon admission, with a structured recording of the patient’s psychopathology (including e.g., information on suicidal ideation) and their social characteristics and an unstructured documentation of their disorder development, biography and family history. In addition, patients are assessed by the treating psychiatrist on admission and discharge with the standardized Global Assessment of Functioning [GAF, a numeric scale used by health professionals to estimate the basic functioning of a subject at a certain time point between 0 and 100 (36)]. During an inpatient stay, clinically relevant information is regularly documented by all medical professionals and the most relevant clinical information is summarized in the patient’s discharge letter, including documentation of the diagnosis, which must always be verified be a psychiatric specialist. Furthermore, a one-time psychological assessment by a clinical psychologist is aimed at for all those patients who are not only inpatient for a crisis intervention for a very short period of time. This includes a detailed exploration of the biography including an assessment of relevant risk factors like ACEs and the use of standardized tests that focus on to the patient’s main disability or diagnostic uncertainty (e.g., personality disorder). While the retrospectively evaluable clinical information from patients is partly based on structured assessments (such as psychopathology and GAF scores), the main information is based on unstructured interviews.

For the assessment of the study potential risk factors identified in previous literature (such as ACEs, sociodemographic or clinical characteristics) were comprehensively extracted from all unstructured and structured patient records created by psychiatrists or psychologists in the context of any psychiatric in- or outpatient treatment. A number of variables relating to ACEs, which were initially collected in great detail (e.g., frequency and duration per ACE), had to be summarized and simplified for this analysis. Thus, based on previous literature, ACEs were assessed for classification into three main categories: family dysfunction (divorce of parents before 18, growing up with one parent, death of parent(s) before 18, adoptive or foster child), neglect (emotional or physical neglect) and abuse (verbal, physical or sexual abuse). Thereafter, the number of ACEs per person was calculated, with and without divorce included. If no statement to a certain ACEs was identified in the records, the specific ACE was rated as not having been present.

In order to assess diagnostic characteristics, all discharge diagnosis of child and adolescent or adult psychiatric admissions at the University Hospital in Tulln were extracted in detail and summarized as ICD-10-group for further evaluation. Furthermore, they were specified as primary diagnosis at last PIT, primary diagnosis during any PIT, secondary diagnosis during any PIT and diagnosis during any PIT. Finally, the number of diagnosed ICD-10-F-subgroups per patient was calculated. For the assessment of the Global Assessment of Functioning documented scores at admission and discharge were used, and the GAF change during an inpatient stay per patient calculated. Patients without GAF scores were excluded from the GAF-score assessment. An inter-rater reliability test had not be conducted. Within further clinical characteristics, any previous suicide attempt was assessed. If available, number of suicide attempts and age at first suicide attempt in years were documented. Suicidal ideation as well as endangerment of self or others was extracted from the patient admission forms described above, as well as information on compliance to medication at time of admission and acceptance of disease. Information regarding relationship problems, was primarily found in clinical psychological assessments and was categorized as given or not given.

To assess relevant inpatient treatment characteristics the hospital IT system provided an overview of all patient’s in- and outpatient treatments at the University Hospital Tulln. This allowed extraction of the variables date of first psychiatric (CAP or adult) admission, date of first psychiatric adult admission and date of last psychiatric adult admission so that the age at the specified admissions as well as the durations between first and last admission as well as the duration first child and adolescent psychiatric admission to first adult psychiatric admission could be calculated. In addition, the total number of psychiatric admissions and the number of child and adolescent or adult psychiatric admissions as well as the maximum duration of inpatient stays could be extracted.

As outpatient care is not provided solely by the hospital but also by outpatient specialists, the current investigation focused on inpatient treatment characteristics only. This study was conducted according to the Helsinki Declaration and was ethically approved by the ethics committee of Lower Austria (No.: *GS1-EK-4/337-2015*). Data collection took place between August 2016 and November 2018.

### Data analyses

Statistical analyses were performed with IBM SPSS Statistics 26 (37). Data was primarily analyzed using descriptive statistics, with interferential statistical methods at a 5% significance level applied as appropriate. Group differences were tested for categorical variables with the Pearson χ^2^ test, if the group size was expected to be above five; otherwise, Fisher’s exact test was used. For non-normally distributed variables, the Mann-Whitney-U-test was applied. Finally, a logistic regression analysis, factoring in sex, was used to group all independent ACE variables and carry out predictive modeling of young adult inpatients with a former CAP admission. Due to the explorative rather than confirmatory nature of this study, the authors abstained from adjustments for multiple testing (38, 39). Therefore, unadjusted *p*-values are given, to remain strictly hypothesis-generating.

## Results

### General sample characteristics

Of all 427 patients that fulfilled the study criteria (having received at least 1 day of psychiatric inpatient treatment at the Department of Adult Psychiatry of the University Hospital Tulln between 2010 and 2015, and who were aged 18–25) 8.7% (*n* = 37) had to be excluded from the investigation due to a lack of data. The exclusion criterium was fulfilled if a detailed description of the clinical picture and existing risk factors (such as ACEs) was missing. Excludes were almost exclusively patients who had once a 1-day inpatient stay. Therefore, the sample comprised 390 patients, with 51.8% (*n* = 202) being female (Table 1). The mean age at first psychiatric inpatient treatment (PIT) was exactly 20 years (SD: 2y1mo) and mean age at last PIT was 20 years, 8 months (SD: 1y10mo). 10.3% (*n* = 40) had previously received inpatient treatment at the local department of Child and Adolescent Psychiatry (CAP-IPs).

**TABLE 1:** Sociodemographic characteristics of the total sample of young adult inpatients (*n*, %) and specifically listed (*n*, %) and compared (Pearson χ^2^) those with a former child and adolescent psychiatric inpatient treatment (former CAP-IPs) and those without a former CAP admission (non-CAP-IPs).

		Total sample		Former CAP-IPs		Non-CAP-IPs		Pearson χ^2a^			
				
		*n*	%	*n*	%	*n*	%	χ^2^	df	*p*	*V*
Total		390		40		350					
Sex	Male	188	48.2	9	22.5	179	51.1	**11.8**	**1**	**0.001**	**0.174**
	Female	202	51.8	31	77.5	171	48.9				
Status of employment	Employed/Apprentice	226	57.9	16	40.0	210	60.0	**5.89**	**1**	**0.015**	**0.123**
	Unemployed/Retired	164	42.1	24	60.0	140	40.0				
	Employed	217	55.6	16	40.0	201	57.4				
	Apprentice	9	2.3	0	0.0	9	2.6				
	Unemployed	134	34.4	20	50.0	114	32.6				
	Early retirement	30	7.7	4	10.0	26	7.4				
	Student/Unclear	0	0.0	0	0.0	0	0.0				
Highest degree of education	Compulsory school	188	48.2	22	55.0	166	47.4	1.40	2	0.496	0.064
	Apprenticeship	74	19.0	5	12.5	69	19.7				
	University-entrance university degree	77	19.7	8	20.0	69	19.7				
	Unclear	51	13.1	5	12.5	46	13.1				
Country of birth	Austria	361	92.8	37	92.5	324	92.8	0.58	2	0.750	0.038
	Germany	5	1.3	1	2.5	4	1.1				
	Other countries	23	5.9	2	5.0	21	6.0				
Status of relationship	Single	368	94.4	38	97.4	330	95.4	0.49	2	0.782	0.036
	Married	14	3.6	1	2.6	13	3.8				
	Separated	3	0.8	0	0.0	3	0.9				

^a^For this and subsequent tables (alphabetical order): df, degree of freedom; *p*, probability value (below 0.05 is regarded as significant and marked in bold); (Pearson) χ^2^, Pearson’s chi-squared test; *V*, Cramer’s V.

### Former child and adolescent psychiatry inpatients are predominantly female and unemployed

Former CAP-IPs were shown to be disproportionally female (77.5%; χ^2^ = 11.80, *V* = 0.174, *p* = 0.001) and have a worse employment status (χ^2^ = 5.89, *V* = 0.123, *p* = 0.015) compared to those without CAP admissions. However, no group difference in highest level of educational attainment or country of birth were found (Table 1).

### Experiences of adoption, foster care and sexual abuse characterize former child and adolescent psychiatry inpatients

Comparison between inpatients with and without former CAP admissions (Table 2) revealed that the former were more likely to have grown up with some form of family dysfunction. Former CAP-IPs had experienced adoption, foster care and becoming orphans significantly more frequently. Furthermore, young adult inpatients with a former CAP admission had more frequent documentation of experiences of neglect, with increased rates of both emotional and physical neglect. Across all forms of abuse, a nearly twofold increase was observed in the former CAP group compared those without former CAP care, with the highest discrepancy for experiences of sexual abuse. Excluding divorce, young inpatients with a former CAP hospitalization had significantly higher total numbers of ACEs compared to those without former CAP care. Furthermore, assessing and comparing the average number of different forms of ACEs per patient revealed that former CAP-IPs had experienced a significantly higher number of diverse ACEs throughout their childhood (mean 2.0 vs 1.1; U: 4,522.5, Z: 3.84, *p* ≤ 0.001, *r* = 0.195).

**TABLE 2:** Comparison of recorded adverse childhood experiences (ACEs; *n*, %, Pearson χ^2^) in young adult inpatients with a former child- and adolescent psychiatric inpatient treatment (former CAP-IPs) and those without (non-CAP-IPs).

ACEs	Former CAP-IPs		non-CAP-IPs		Pearson χ^2a^			
			
	*n*	%	*n*	%	χ^2^	df	*p*	*V*
Family dysfunction	28	**70.0**	**151**	**43.1**	**10.43**	**1**	**0.001**	**0.164**

Adoptive or foster child	**9**	**22.5**	**14**	**4.0**	**22.14**	**1**	**< 0.001**	**0.238**
Death of parent(s) before 18	**5**	**12.5**	**17**	**4.9**	**3.94**	**1**	**0.047**	**0.101**
Growing up with one parent	10	25.0	52	14.9	2.76	1	0.097	0.084
Divorce of parents before 18	13	32.5	106	30.3	0.08	1	0.773	0.015
Neglect	**20**	**50.0**	**87**	**24.9**	**11.40**	**1**	**0.001**	**0.171**
Emotional neglect	**19**	**47.5**	**84**	**24.0**	**10.2**	**1**	**0.001**	**0.162**
Physical neglect	**4**	**10.0**	**6**	**1.7**	**9.86**	**1**	**0.002**	**0.159**
Abuse	**16**	**40.0**	**74**	**21.1**	**7.19**	**1**	**0.007**	**0.136**
Sexual abuse	**11**	**27.5**	**20**	**5.7**	**23.29**	**1**	**< 0.001**	**0.244**
Verbal abuse	5	12.5	24	6.9	1.66	1	0.198	0.065
Physical abuse	6	15.0	47	13.4	0.08	1	0.784	0.014
ACEs (total)	30	75.0	214	61.1	2.94	1	0.086	0.087
ACEs (total) excl. divorce	**27**	**67.5**	**163**	**46.6**	**6.29**	**1**	**0.012**	**0.127**

Bold values represent the significant results.

Further logistic regression analysis of ACEs (Table 3) including sex as a covariate, revealed that those who had experienced adoption or foster care were four and half times (OR: 4.5; 95% CI: 1.4/14.4) more likely to have been admitted to CAP before, and those who had experienced sexual abuse (OR: 3.0; 95% CI: 1.1/8.2) were three times more likely. Both aforementioned factors could be identified as the primary ACE risk factors for CAP admissions. Females were three times (OR: 3.04; CI: 1.29/7.20) more likely to be hospitalized in childhood than males. The model itself was statistically significant (Pearson’s χ^2^ (10): 44.24, *p* ≤ 0.001), explained 22.2% (Nagelkerke *R*^2^) of the variance, and correctly classified 89.7% of cases.

**TABLE 3:** Logistic regression model for recorded adverse childhood experiences and the co-variable sex as predictor for a former child and adolescent inpatient treatment in young psychiatric inpatients.

Independent variables	B	S.E.	Wald	df	*p*	OR	95% CI LB/UB
Sex	**1.113**	**0.439**	**6.433**	**1**	**0.011**	**3.04**	**1.29/7.20**
Adoptive or foster child	**1.507**	**0.59**	**6.519**	**1**	**0.011**	**4.51**	**1.42/14.36**
Sexual abuse	**1.084**	**0.52**	**4.342**	**1**	**0.037**	**2.96**	**1.07/8.19**
Emotional neglect	0.803	0.41	3.833	1	0.05	2.23	1.00/4.99
Physical neglect	1.382	0.825	2.804	1	0.094	3.98	0.79/20.06
Death of parent(s) before 18	1.132	0.749	2.285	1	0.131	3.10	0.72/13.46
Physical abuse	–0.61	0.55	1.234	1	0.267	0.54	0.19/1.60
Divorce of parents before 18	0.279	0.428	0.424	1	0.515	1.32	0.57/3.06
Growing up with only one parent	0.252	0.558	0.204	1	0.652	1.29	0.43/3.84
Verbal abuse	0.100	0.636	0.025	1	0.876	1.11	0.32/3.84
Constant	–3.687	0.437	71.041	1	<0.001	0.025	

B, coefficients; CI, Confidence interval; df, degree of freedom; LB, lower bound; OR, odds ratio; *p*, probability value [<0.05 is regarded as significant (bold)]; S.E., standard error; UB, upper bound; Wald, Wald chi-square test.

### Comorbidity and suicide attempts are more common in former child and adolescent psychiatry inpatients

To examine diagnostic characteristics including cross-category comorbidities, every primary and secondary ICD-10 diagnosis, from each psychiatric admission from childhood to young adulthood, was taken and grouped into sub-categories (e.g., F00.0–F09 as F0). The results showed that, young adult inpatients with former CAP inpatient treatment more frequently suffered from a psycho-somatic (primarily eating-, F5) or personality and behavioral (F6) disorder at the most recent PIT compared to those who had not been hospitalized before in CAP (Table 4). Furthermore, across all admissions, they were more frequently diagnosed with a primary or secondary diagnosis of a mood (F3), neurotic, stress-related and somatoform (F4), psychosomatic (F5), personality and behavioral (F6), or neurodevelopmental disorder; similarly, rates of intellectual disability (F7) and behavioral and emotional disorders with onset usually occurring in childhood and adolescence (F9) were higher (Table 5). As expected, former CAP-IPs were burdened by more frequent cross-category comorbidity, with an average of three main psychiatric diagnostic categories compared to two in non-CAP-IPs (U: 3,222.0, *Z* = –5.99, *p* ≤ 0.001, *r* = –0.303).

**TABLE 4:** Comparison of the primary ICD-10 diagnosis received at last psychiatric inpatient treatment (*n*, %, Pearson χ^2^) in young adult inpatients with a former child- and adolescent psychiatric inpatient treatment (former CAP-IPs) and those without it (non-CAP-IPs).

Disorders (ICD-10)	Former CAP-IPs		Non-CAP-IPs		Pearson χ^2^			
			
	*n*	%	*n*	%	χ^2^	df	*p*	*V*
Organic (F0)	0	0.0	2	0.6	0.230	1	1.0^a^	0.024
Substance use (F1)	4	10.0	45	12.9	0.267	1	0.802^a^	0.026
Schizophrenia, schizotypal and delusional (F2)	2	5.0	53	15.1	3.049	1	0.081	0.088
Mood (F3)	12	30.0	134	38.3	1.052	1	0.305	0.052
Neurotic, stress-related and somatoform (F4)	7	17.5	65	18.6	0.027	1	0.869	0.008
Psycho-somatic (F5)	**3**	**7.5**	**4**	**1.1**	**8.230**	**1**	**0.026** ^a^	**0.145**
Personality and behavioral (F6)	**9**	**22.5**	**36**	**10.3**	**5.247**	**1**	**0.033** ^a^	**0.116**
Intellectual disability (F7)	1	2.5	3	0.9	0.954	1	0.352^a^	0.049
Psychological development (F8)	1	2.5	0	0.0	8.77	1	0.103^a^	0.150
Behavioral and emotional disorders with onset in childhood and adolescence (F9)	0	0.0	1	0.3	0.115	1	1.0^a^	0.017
Somatic and Z	1	2.5	7	2.0	0.045	1	0.583^a^	0.011

^a^Fisher’s exact test. Bold values represent the significant results.

**TABLE 5:** Comparison of the primary and secondary ICD-10 diagnosis received at any psychiatric inpatient treatment (*n*, %, Pearson χ^2^) in young adult inpatients with a former child- and adolescent psychiatric inpatient treatment (former CAP-IPs) and those without it (non-CAP-IPs).

Disorders (ICD-10)	Former CAP-IPs		Non-CAP-IPs		Pearson χ^2^			
			
	*n*	%	*n*	%	χ^2^	df	*p*	*V*
Organic (F0)	1	2.5	5	1.4	0.27	1	0.480^a^	0.026

Substance use (F1)	16	40.0	155	44.3	0.27	1	0.605	0.026
Schizophrenia, schizotypal and delusional (F2)	3	7.5	60	17.1	2.46	1	0.116	0.079
Mood (F3)	**27**	**67.5**	**174**	**49.7**	**4.55**	**1**	**0.033**	**0.108**
Neurotic, stress-related and somatoform (F4)	**28**	**70.0**	**113**	**32.3**	**20.12**	**1**	**<0.001**	**0.238**
Psycho-somatic (F5)	**8**	**20.0**	**21**	**6.0**	**10.22**	**1**	**0.005^a^**	**0.162**
Personality and behavioral (F6)	**17**	**42.5**	**49**	**14.0**	**20.74**	**1**	**<0.001**	**0.231**
Intellectual disability (F7)	**4**	**10.0**	**5**	**1.4**	**11.70**	**1**	**0.008^a^**	**0.173**
Psychological development (F8)	1	2.5	3	0.9	0.95	1	0.352^a^	0.049
Behavioral and emotional disorders with onset in childhood and adolescence (F9)	**7**	**17.5**	**7**	**2.0**	**24.92**	**1**	**<0.001^a^**	**0.253**
Somatic and Z	1	2.5	5	1.4	0.46	1	0.50	0.034

^a^Fisher’s exact test. Bold values represent the significant results.

A lower frequency of problematic cannabinoid use (χ^2^: 5.77, *V* = 0.122, *p* = 0.016), but similar levels of problematic use of alcohol (χ^2^: 0.48, *V* = 0.035, *p* = 0.487), multiple drugs (χ^2^: 1.64, *V* = 0.065, *p* = 0.201) or nicotine (χ^2^: 2.05, *V* = 0.072, *p* = 0.153), was observed in former CAP-IPs compared to non-CAP-IPs. In addition, former CAP-IPs had greater impairments in their daily functioning (GAF) at admission (U: 1,900.0, *Z* = –3.01, *p* = 0.003, *r* = –0.200) and discharge (U: 1,864.5, *Z* = –2.79, *p* = 0.005, *r* = –0.187) at the most recent psychiatric admission. This was reflected in a median GAF, which was ten points lower at both times (Mdn: admission 30 vs 40, discharge 50 vs 60). Although both cohorts showed a similar improvement throughout inpatient care (U: 2,517.5, *Z* = 0.63, *p* = 0.527, *r* = 0.043), former CAP-IPs remained in hospital for twice as long (53 vs 20.7 days).

A higher rate of suicide attempts was identified in the former CAP inpatients (χ^2^: 5.01, *V* = 0.113, *p* = 0.025), with documented suicide attempts for half (50%) of this group, compared to approximately a third (32.2%) of those without former CAP admissions. In comparison to non-CAP-IPs, former CAP-IPs made their first suicide attempt on average 1.5 years earlier in development and reported more than twice as many attempts (0.6 vs 1.5). Similarly, former CAP-IPs were more frequently recorded as endangering themselves or others at admission (χ^2^: 11.84, *V* = 0.174, *p* = 0.001), and experienced more relationship problems (χ^2^: 9.63, *V* = 0.157, *p* = 0.002). Although acceptance of their diagnosis did not differ between the two cohorts (χ^2^: 2.34, *V* = 0.078, *p* = 0.310), former CAP-IPs were less compliant with their medication (χ^2^: 11.42, *V* = 0.172, *p* = 0.003).

### Former child and adolescent psychiatry inpatients are admitted twice as frequently in young adulthood

The results show that the average age for first CAP admission was approximately 16 years (M: 16.1, SD: 1.4). After an average of two additional CAP admissions, patients were admitted to an adult psychiatric ward for the first time at approximately 19 years of age (M: 19.1, SD: 1.2) and received an average of 3.4 (SD: 4.3) further admissions. Compared to inpatients without former CAP admissions, those with additional former CAP care were admitted in adulthood twice as often (U: 4,435, *Z* = –462, *p* ≤ 0.001, *r* = –0.234) and had a 2.5 times longer maximum duration of PIT in adulthood (U: 3,569, *Z* = –5.08, *p* ≤ 0.001, *r* = –0.257).

## Discussion

The current study aimed to determine risk factors for former admission with Child and Adolescent Psychiatry (CAP) in a representative sample of young adult inpatients, with a particular focus on adverse childhood experiences (ACEs). Our investigation revealed several key findings. First, the ten percent of all 390 young adult inpatients that were former CAP inpatients were predominantly female (78%), and either unemployed or in early retirement (60%). Female sex tripled the risk of childhood admissions. Second, ACEs were reported in two thirds of former CAP inpatients, with significantly higher rates of family dysfunction, neglect, and abuse. Of note, sexual abuse and adoption or foster care were identified as the main ACEs that are risk factors for this cohort. Ancillary findings revealed that young adult inpatients who were former CAP inpatients were shown to suffer from psychosomatic or personality disorders, comorbidities and functional impairment, at a higher rate than those without prior CAP admission. Finally, former CAP inpatients were twice as likely to be readmitted into psychiatric care during young adulthood.

These findings build upon prior research highlighting the role of ACEs as a risk factor for mental health problems, particularly in vulnerable groups (14, 15, 40, 41), and help characterize for the first time the unique risk associated with psychiatric readmission across childhood, adolescence, and young adulthood. As there are no prior studies investigating ACEs in young adults with former CAP admissions, our findings can be best compared to studies assessing CAP cohorts. Rytilä-Manninen et al. (7) found that 85% of 13–17-year-old inpatients reported at least one adverse life event, 60% reported two or more, and 21% reported four or more, approximately four times the rate (2.2 vs 0.6) compared to the average population. Thus, the average frequency of two ACEs reported by the presently studied former CAP inpatients, appears to be comparable to that of CAP inpatients in previous literature. Higher rates of ACE co-occurrence are known to be associated with worse health outcomes, with evidence that psychological distress can be better predicted by the total number of ACEs than by specific ACEs (14, 15, 42, 43). Based on this, the present cohort of former CAP inpatients appears to be at particular risk. While young adult inpatients with a former CAP admission (compared to those without a CAP admission) experienced significantly higher rates of (i) adoption and foster care, (ii) orphancy, (iii & iv) emotional and physical forms of negligence, as well as (v) sexual abuse, the logistic regression model controlling for sex only identified sexual abuse (OR, 3.0) and experiences of adoption and foster care (OR, 4.5) as significant risk factors. In a medical history study of approximately 1,400 CAP inpatients, a similar proportion of patients had experienced incidents of documented abuse (36 vs 40% in the current study), and physical and sexual harm each increased the likelihood of gaining at least one additional diagnosis, leading to cross-category comorbidity. Sexual abuse was also associated with longer hospital admissions (30). Further studies of CAP inpatients demonstrate the serious impact sexual abuse can have on the lives of children and adolescents, with observations of a twofold greater risk of non-suicidal self-injury and suicide attempts (40) as well as an increased likelihood of developing internalizing or externalizing disorders (7). These observations are in line with numerous studies reporting an increased risk across a broad range of mental health problems and disorders, including depression, post-traumatic stress disorder, and anxiety disorders (7, 44–46).

Growing up adopted or in a foster family was another ACE associated with former CAP admissions for young adult inpatients in our study, aligning with numerous studies that emphasizing the multitude of burdens that adopted or fostered children face (47–49). Negative health outcomes identified by these studies, such as lower social functioning, increased rates of substance use disorders, mood or anxiety disorders, and increased self-destructive behaviors could explain the increased rates and length of psychiatric inpatient treatment observed for adopted or foster children in the current study (47, 49–51). Furthermore, the quantity of ACEs could exacerbate risk in this population, as a recent study showed that number of ACEs predicted externalizing or internalizing problems in adopted children (52).

Two further findings from the ACE regression model described above are worth being discussed. On the one hand, the almost significant negative impact of emotional neglect (OR = 2.2) and on the other hand, the apparently protective effect of physical abuse (OR = 0.5) in those young adult inpatients with former CAP admissions. While the negative effects of emotional neglect on mental health and its care are well-studied (53–55), the apparent protective effects of physical abuse experiences contradicts previous findings (20, 56). Since we controlled for sex in our ACE model, the effect found cannot be explained solely by the fact that men experience more physical abuse in childhood than women (57). However, the finding could be interpreted on the one hand as a result of possible underreporting (also by the parents) due to fear of legal consequences, and on the other hand in the sense of the protective function of early inpatient child and youth care. Since these considerations are speculative, it would be useful for future research to address these questions.

As expected, young inpatients with a former CAP admission were burdened with higher cross-category comorbidity, with an average of nearly three diagnostic categories across all psychiatric inpatient treatments. Increased rates of comorbidity in patients with former CAP treatment have been previously reported in studies examining the transition from CAP to adult psychiatric care (34, 58). Furthermore, diagnostic change during this service transition – which has been observed to occur in three quarters of CAP inpatients in a recent study in Wales (58) – could have contributed to the increased rates of cross-categorical comorbidity in the current study, which assessed admissions spanning childhood, adolescence and young adulthood.

Specifically, former CAP inpatients had higher rates of psychosomatic or personality disorder at their most recent PIT, and across all admissions had higher rates of being diagnosed with a mood neurotic, stress-related and somatoform-, psychosomatic, or personality disorder, as well as behavioral and emotional disorders with onset usually occurring in childhood and adolescence and intellectual disability. Contrary to expectations and previous findings, the current cohort of former CAP inpatients did not suffer from greater rates of schizophrenia spectrum disorders, affective or pervasive developmental disorders (32, 59). The finding of increased rates of personality disorders for young adult inpatients with a former CAP admission is, however, in line with previous research (32). One follow-up study of former CAP patients reported observations of nearly twice the rate of any current ICD diagnosis and quadruple the rates of personality disorders (13%) compared to the general population, approximately 13 years after discharge (6). In our study, the prevalence rate for personality disorders was even higher, with the rate reported at the most recent admission almost double again (23%) and the rate across all admissions three times as high (43%) (6). Increased rates of personality disorders, primarily based on the borderline type, in young adult inpatients with former CAP care, could also be discussed in the context of a higher exposure to ACEs found in this group, which are known to influence neurocognitive change and the development of personality disorders (54). Likewise, the increased rates of psychosomatic disorders, mainly eating disorders from the anorexia nervosa type could be discussed, since increased rates of ACE have been repeatedly found in those with eating disorders (60, 61). However, it must not be forgotten that anorexia nervosa is one of the most serious chronic mental disorders with an early onset and the highest mortality rate and therefore requiring reoccurring inpatient care (62, 63).

In addition to the aforementioned group differences, the young adult inpatients with former CAP admissions from the current study were also more severely functionally impaired and more frequently a danger to themselves, with more than twice as many suicide attempts as inpatients without former CAP admissions. These observations may be explained by high rates of personality and neurodevelopmental disorders in this cohort. Additionally, the high rate of adverse life events could play an important role, as ACEs have been shown to increase the likelihood of (self-)risk behavior and suicide attempts (41, 64, 65).

Although the full sample of 390 inpatients was characterized by an almost even sex distribution (52% females), female patients represented the majority (78%) of young adult inpatients with a former CAP admission. This finding is in line with several previous papers showing a greater use of psychiatric services in women (1, 26). While one Finnish cohort study of adolescent CAPs found a similar sex distribution (71% female) (7), the current results contradict observations from large cohort studies with around 1,000 participants of a balanced sex distribution (30, 33) or a predominantly male population (8). However, this difference may be due to the different methods: whereas Gyllenberg et al. (8) focused on childhood psychopathology and conduct disorders predicting hospitalization in the adolescence and young adulthood, the present study had a retrospective view and concentrated on former CAP treatment of young adult inpatients. Unfortunately, comparison with the local CAP cohort could not be carried out in the current study due to limitations of the ethical permission/approval. In order to better understand the role of sex in relation to characteristics found of young adult inpatients with former CAP admissions, a corresponding analysis would be of great relevance. However, since the number of male patients with former CAP admissions in our study was only nine, a serious statistical comparison (with the 31 females) was unfortunately not expedient. Future studies, however, should plan for a sufficient cohort size to conduct such analysis, since any observed differences might also be impacted by corresponding sex differences in diagnostic practices, prevalence and differences in help-seeking behavior.

Despite the contribution of the current study, there are several limitations particularly due to the explorative and retrospective design worth noting. First, it is likely that ACEs were underreported due to the reliance on retrospective assessment of medical records that were based on unstandardized clinical assessments. Related to this, the counting and categorization of ACEs cannot claim to fully capture the complex burden of these experiences, which are known to often overlap and reoccur (66, 67). To ensure the best quality of data given these limitations, data collection was done by a team with experience in mental health and assessed based on predefined variables; however, an inter-rater reliability test had not been done. Secondly, due to the restrictions of the ethical approvals, a longitudinal follow up of all (CAP) inpatients was not possible and only care at the University Hospital Tulln could be assessed, limiting the information available regarding patients’ general health service use. Therefore, patients who may have moved to another state would be missed in the analysis, also due to a lack of electronic medical records that were available for assessment at the national level. Nevertheless, due to the clearly regulated regional allocation of hospital care, our findings should still convey a representative picture of the people in greatest need of care.

In conclusion, the present study found that ACEs, particularly sexual abuse and being fostered or adopted, were significantly more common in young adult inpatients with former psychiatric admissions in childhood and adolescence then in those without former CAP inpatient care. Higher rates of severe ACEs in patients with early and recurrent psychiatric care could be understood and interpreted in the following ways, which might coexist and influence each other. First, the experience of profound adverse events early in life might lead to earlier mental health problems due to its well described negative “toxic stress” (20) and, accordingly, to earlier and more frequent treatment. Most relevant seem ACEs that have a potentially high negative impact on secure attachment relationships and possibly a correspondingly high impact on the stress response system. Second, it could be understood based on the environment in which those affected by ACEs grow up and find themselves. Thus, the experience of more unstable relationships, limited social and financial resources, and consequently appropriate support, can lead not only to maladaptive pathways with negative effects on individual psychological well-being, but also to an increased need for external support and psychiatric care (27–29). Further characteristics observed in those young inpatients with former CAP admissions, such as increased rates of psychosomatic or personality disorders, clinical disabilities or unemployment, could also be associated to the previous explanation and warrant further investigation. The negative effects of unemployment on mental and physical health may be greater at a younger age (68, 69), particularly as young adults are challenged with finding their role in society and gaining autonomy (70–72). Therefore, it seems necessary to further investigate the role of employment as a preventive factor for recurrent or prolonged admissions, and special attention should be paid to improving the educational and employment opportunities in young psychiatric patients. Although a systematic examination of individual and cumulative ACEs has limitations (particularly applied to a general population), the clinical implication of this study is to highlight the necessity of improving our understanding of adverse events as risk factors for health and service care outcomes (73). This may help ensure appropriate treatment is accessible in child psychiatry setting in order to reduce further sequelae of childhood traumatization or other hardships, as there is evidence for inpatient therapeutic group treatment reducing PTSD and anxiety symptoms and improving self-esteem in traumatized adolescent girls (74). Since ACEs have been shown to increase the risk of future sexual and general revictimization (67), the integration and further improvement of interventions for ACE appears to be of particular importance for young inpatients with recurrent or extended admissions. A specific focus on family relationships and problem-solving skills could be of benefit (75). Finally, future investigations should explore the role of (early) outpatient- or community therapy as preventive strategies to not only avoid subsequent heavy use of mental health services, but also to evaluate the patient’s social context (meaningful relationships, work, family) and social skills in order to improve our understanding of heavy service use.

## Data availability statement

The data analyzed in this study is subject to the following licenses/restrictions: The data are not publicly available due to privacy and ethical restrictions. Requests to access these datasets should be directed to MF, matthaeus.fellinger@meduniwien.ac.at.

## Ethics statement

The study involving human participants was reviewed and approved by Ethics Committee of Lower Austria- No. GS1-EK-4/337-2015. Due to the retrospective design, written informed consent from the participants’ legal guardian/next of kin was not required to participate in this study in accordance with the national legislation and the institutional requirements.

## Author contributions

MF and MA: conceptualization and methodology. MF and KK-B: statistics. MF: writing—original draft preparation. MF, MA, and PK: project administration. All authors read and agreed to the published version of the manuscript.

## Abbreviations

ACE: adverse childhood experiences
CAP: child and adolescent psychiatry
CAP-IPs: child and adolescent psychiatry inpatients
GAF: global assessment of functioning
PIT: psychiatric inpatient treatment.

## References

[1] Astrup H Myhre MØ Kildahl AT Walby FA. Suicide after contact with child and adolescent mental health services—a national registry study. *Front Psychiatry.* (2022) 13:886070. 10.3389/fpsyt.2022.886070 35615447 PMC9124860

[2] Horwitz SM Storfer-Isser A Demeter C Youngstrom EA Frazier TW Fristad MA Use of outpatient mental health services among children of different ages: are younger children more seriously ill? *Psychiatr Serv.* (2014) 65:1026–33. 10.1176/appi.ps.201300209 24789735 PMC4121963

[3] Knapp M McCrone P Fombonne E Beecham J Wostear G. The maudsley long-term follow-up of child and adolescent depression: 3. impact of comorbid conduct disorder on service use and costs in adulthood. *Br J Psychiatry.* (2002) 180:19–23. 10.1192/bjp.180.1.19 11772846

[4] Hofstra MB Van der Ende J Verhulst FC. Continuity and change of psychopathology from childhood into adulthood: a 14-year follow-up study. *J Am Acad Child Adolesc Psychiatry.* (2000) 39:850–8. 10.1097/00004583-200007000-00013 10892226

[5] Kim-Cohen J Caspi A Moffitt TE Harrington H Milne BJ Poulton R. Prior juvenile diagnoses in adults with mental disorder: developmental follow-back of a prospective-longitudinal cohort. *Arch Gen Psychiatry.* (2003) 60:709–17. 10.1001/archpsyc.60.7.709 12860775

[6] Brieger P Blöink R Sommer S Marneros AA. Catch-up study of former child and adolescent psychiatric inpatients: psychiatric status in adulthood. *Psychopathology.* (2001) 34:43–9. 10.1159/000049279 11150930

[7] Rytilä-Manninen M Lindberg N Haravuori H Kettunen K Marttunen M Joukamaa M Adverse childhood experiences as risk factors for serious mental disorders and inpatient hospitalization among adolescents. *Child Abuse Negl.* (2014) 38:2021–32. 10.1016/j.chiabu.2014.10.008 25455961

[8] Gyllenberg D Sourander A Niemelä S Helenius H Sillanmäki L Piha J Childhood predictors of later psychiatric hospital treatment: findings from the finnish 1981 birth cohort study. *Eur Child Adolesc Psychiatry.* (2010) 19:823–33. 10.1007/s00787-010-0129-1 20821264

[9] Sourander A Helenius H Piha J. Parent and teacher reports of problem behaviors in child psychiatric inpatients: cross-informant correlations on admission and at 5-month follow-up. *Child Psychiatry Hum Dev.* (1995) 26:85–95. 10.1007/BF02353233 8565650

[10] Pfeiffer SI Strzelecki SC. Inpatient psychiatric treatment of children and adolescents: a review of outcome studies. *J Am Acad Child Adolesc Psychiatry.* (1990) 29:847–53. 10.1097/00004583-199011000-00001 2273010

[11] Green J Jacobs B Beecham J Dunn G Kroll L Tobias C Inpatient treatment in child and adolescent psychiatry – a prospective study of health gain and costs. *J Child Psychol Psychiatry.* (2007) 48:1259–67. 10.1111/j.1469-7610.2007.01802.x 18093032

[12] Thornicroft G. Most people with mental illness are not treated. *Lancet.* (2007) 370:807–8. 10.1016/S0140-6736(07)61392-0 17826153

[13] Bardach NS Coker TR Zima BT Murphy JM Knapp P Richardson LP Common and costly hospitalizations for pediatric mental health disorders. *Pediatrics.* (2014) 133:602–9. 10.1542/peds.2013-3165 24639270 PMC3966505

[14] Petruccelli K Davis J Berman T. Adverse childhood experiences and associated health outcomes: a systematic review and meta-analysis. *Child Abuse Negl.* (2019) 97:104127. 10.1016/j.chiabu.2019.104127 31454589

[15] Hughes K Bellis MA Hardcastle KA Sethi D Butchart A Mikton C The effect of multiple adverse childhood experiences on health: a systematic review and meta-analysis. *Lancet Public Health.* (2017) 2:e356–66. 10.1016/S2468-2667(17)30118-4 29253477

[16] Struck S Stewart-Tufescu A Asmundson AJN Asmundson GGJ Afifi TO. Adverse childhood experiences (ACEs) research: a bibliometric analysis of publication trends over the first 20 years. *Child Abuse Negl.* (2021) 112:104895. 10.1016/j.chiabu.2020.104895 33388607

[17] Leboyer M Berk M Yolken RH Tamouza R Kupfer D Groc L. Immuno-psychiatry: an agenda for clinical practice and innovative research. *BMC Med.* (2016) 14:173. 10.1186/s12916-016-0712-5 27788673 PMC5084344

[18] Prentice DM Otaibi BW Stetter C Kunselman AR Ural SH. The association between adverse childhood experiences and postpartum depression. *Front Glob Womens Health.* (2022) 3:898765. 10.3389/fgwh.2022.898765 35692946 PMC9183059

[19] Bozzatello P Rocca P Baldassarri L Bosia M Bellino S. The role of trauma in early onset borderline personality disorder: a biopsychosocial perspective. *Front Psychiatry.* (2021) 12:721361. 10.3389/fpsyt.2021.721361 34630181 PMC8495240

[20] Anda RF Felitti VJ Bremner JD Walker JD Whitfield C Perry BD The enduring effects of abuse and related adverse experiences in childhood. A convergence of evidence from neurobiology and epidemiology. *Eur Arch Psychiatry Clin Neurosci.* (2006) 256:174–86. 10.1007/s00406-005-0624-4 16311898 PMC3232061

[21] Garner AS Shonkoff JP Committee on Psychosocial Aspects of Child and Family Health [CPACFH], Committee on Early Childhood, Adoption, and Dependent Care. Early childhood adversity, toxic stress, and the role of the pediatrician: translating developmental science into lifelong health. *Pediatrics.* (2012) 129:e224–31. 10.1542/peds.2011-2662 22201148

[22] Haahr-Pedersen I Hyland P Hansen M Perera C Spitz P Bramsen RH Patterns of childhood adversity and their associations with internalizing and externalizing problems among at-risk boys and girls. *Child Abuse Negl.* (2021) 121:105272. 10.1016/j.chiabu.2021.105272 34438263

[23] Assink M van der Put CE Meeuwsen MWCM de Jong NM Oort FJ Stams GJJM Risk factors for child sexual abuse victimization: a meta-analytic review. *Psychol Bull.* (2019) 145:459–89. 10.1037/bul0000188 30777768

[24] Gajos JM Miller CR Leban L Cropsey KL. Adverse childhood experiences and adolescent mental health: understanding the roles of gender and teenage risk and protective factors. *J Affect Disord.* (2022) 314:303–8. 10.1016/j.jad.2022.07.047 35896138 PMC10840483

[25] Gajos JM Leban L Weymouth BB Cropsey KL. Sex differences in the relationship between early adverse childhood experiences, delinquency, and substance use initiation in high-risk adolescents. *J Interpers Violence.* (2022). [Epub ahead of print]. 10.1177/08862605221081927 35466765

[26] Walby FA Myhre MØ Kildahl AT. Contact with mental health services prior to suicide: a systematic review and meta-analysis. *Psychiatr Serv.* (2018) 69:751–9. 10.1176/appi.ps.201700475 29656710

[27] Walsh D McCartney G Smith M Armour G. Relationship between childhood socioeconomic position and adverse childhood experiences (ACEs): a systematic review. *J Epidemiol Community Health.* (2019) 73:1087–93. 10.1136/jech-2019-212738 31563897 PMC6872440

[28] Haugland SH Dovran A Albaek AU Sivertsen B. Adverse childhood experiences among 28,047 norwegian adults from a general population. *Front Public Health.* (2021) 9:711344. 10.3389/fpubh.2021.711344 34381754 PMC8350119

[29] Masten AS. Resilience in children threatened by extreme adversity: frameworks for research, practice, and translational synergy. *Dev Psychopathol.* (2011) 23:493–506. 10.1017/S0954579411000198 23786691

[30] Keeshin BR Strawn JR Luebbe AM Saldaña SN Wehry AM DelBello MP Hospitalized youth and child abuse: a systematic examination of psychiatric morbidity and clinical severity. *Child Abuse Negl.* (2014) 38:76–83. 10.1016/j.chiabu.2013.08.013 24041456

[31] Steinhausen HC Meier M Angst J. The zurich long-term outcome study of child and adolescent psychiatric disorders in males. *Psychol Med.* (1998) 28:375–83. 10.1017/S0033291797005989 9572094

[32] Stulz N Bielinski D Junghan UM Hepp U. [Heavy use of psychiatric hospitals and the use of outpatient services in Switzerland]. *Psychiatr Prax.* (2012) 39:332–8. 10.1055/s-0032-1305231 23044847

[33] Fuchs M Kemmler G Steiner H Marksteiner J Haring C Miller C Child and adolescent psychiatry patients coming of age: a retrospective longitudinal study of inpatient treatment in Tyrol. *BMC Psychiatry.* (2016) 16:225. 10.1186/s12888-016-0910-x 27391233 PMC4938986

[34] Stagi P Galeotti S Mimmi S Starace F Castagnini AC. Continuity of care from child and adolescent to adult mental health services: evidence from a regional survey in Northern Italy. *Eur Child Adolesc Psychiatry.* (2015) 24:1535–41. 10.1007/s00787-015-0735-z 26141538

[35] Bonnie RJ Stroud C Breiner H Committee on Improving the Health [CIH], Board on Children [BC], Institute of Medicine [IM] *Young Adults in the 21st Century.* Washington, DC: National Academies Press (2015). 25855847

[36] Endicott J Spitzer RL Fleiss JL Cohen J. The global assessment scale. A procedure for measuring overall severity of psychiatric disturbance. *Arch Gen Psychiatry.* (1976) 33:766–71. 10.1001/archpsyc.1976.01770060086012 938196

[37] IBM Corp. *IBM SPSS Statistics for Windows, Version 26.0.* Armonk, NY: IBM (2019).

[38] Rothman KJ. No adjustments are needed for multiple comparisons. *Epidemiol Camb Mass.* (1990) 1:43–6. 10.1097/00001648-199001000-00010 2081237

[39] Bender R Lange S. Adjusting for multiple testing—when and how? *J Clin Epidemiol.* (2001) 54:343–9. 10.1016/S0895-4356(00)00314-0 11297884

[40] Isohookana R Riala K Hakko H Räsänen P. Adverse childhood experiences and suicidal behavior of adolescent psychiatric inpatients. *Eur Child Adolesc Psychiatry.* (2013) 22:13–22. 10.1007/s00787-012-0311-8 22842795

[41] Sahle BW Reavley NJ Li W Morgan AJ Yap MBH Reupert A The association between adverse childhood experiences and common mental disorders and suicidality: an umbrella review of systematic reviews and meta-analyses. *Eur Child Adolesc Psychiatry.* (2021) 31:1489–99. 10.1007/s00787-021-01745-2 33638709

[42] Baldwin JR Caspi A Meehan AJ Ambler A Arseneault L Fisher HL Population vs individual prediction of poor health from results of adverse childhood experiences screening. *JAMA Pediatr.* (2021) 175:385–93. 10.1001/jamapediatrics.2020.5602 33492366 PMC7835926

[43] Hunt TKA Slack KS Berger LM. Adverse childhood experiences and behavioral problems in middle childhood. *Child Abuse Negl.* (2017) 67:391–402. 10.1016/j.chiabu.2016.11.005 27884508 PMC5436949

[44] Adams J Mrug S Knight DC. Characteristics of child physical and sexual abuse as predictors of psychopathology. *Child Abuse Negl.* (2018) 86:167–77. 10.1016/j.chiabu.2018.09.019 30308347 PMC6289670

[45] Dias A Sales L Mooren T Mota-Cardoso R Kleber R. Child maltreatment, revictimization and post-traumatic stress disorder among adults in a community sample. *Int J Clin Health Psychol.* (2017) 17:97–106. 10.1016/j.ijchp.2017.03.003 30487885 PMC6220916

[46] Mandelli L Petrelli C Serretti A. The role of specific early trauma in adult depression: a meta-analysis of published literature. childhood trauma and adult depression. *Eur Psychiatry.* (2015) 30:665–80. 10.1016/j.eurpsy.2015.04.007 26078093

[47] Kerker BD Dore MM. Mental health needs and treatment of foster youth: barriers and opportunities. *Am J Orthopsychiatry.* (2006) 76:138–47. 10.1037/0002-9432.76.1.138 16569139

[48] Lehmann S Breivik K Monette S Minnis H. Potentially traumatic events in foster youth, and association with DSM-5 trauma- and stressor related symptoms. *Child Abuse Negl.* (2020) 101:104374. 10.1016/j.chiabu.2020.104374 31982843

[49] Oswald SH Heil K Goldbeck L. History of maltreatment and mental health problems in foster children: a review of the literature. *J Pediatr Psychol.* (2010) 35:462–72. 10.1093/jpepsy/jsp114 20007747

[50] McDonald TP Others. *Assessing the Long-Term Effects of Foster Care: A Research Synthesis.* Washington, DC: Child Welfare League of America, Inc (1996).

[51] Barth RP. On their own: the experiences of youth after foster care. *Child Adolesc Soc Work J.* (1990) 7:419–40. 10.1007/BF00756380

[52] Paine AL Fahey K Anthony RE Shelton KH. Early adversity predicts adoptees’ enduring emotional and behavioral problems in childhood. *Eur Child Adolesc Psychiatry.* (2021) 30:721–32. 10.1007/s00787-020-01553-0 32468437 PMC8060221

[53] Salokangas RKR Schultze-Lutter F Schmidt SJ Pesonen H Luutonen S Patterson P Childhood physical abuse and emotional neglect are specifically associated with adult mental disorders. *J Ment Health.* (2020) 29:376–84. 10.1080/09638237.2018.1521940 30675805

[54] Estric C Calati R Lopez-Castroman J. Adverse childhood experiences and neurocognition in borderline personality disorder: a call-to-action perspective review. *Harv Rev Psychiatry.* (2022) 30:248–60. 10.1097/HRP.0000000000000344 35849742

[55] Brown RC Heines S Witt A Braehler E Fegert JM Harsch D The impact of child maltreatment on non-suicidal self-injury: data from a representative sample of the general population. *BMC Psychiatry.* (2018) 18:181. 10.1186/s12888-018-1754-3 29884152 PMC5994090

[56] Salzinger S Rosario M Feldman RS Ng-mak DS. Adolescent suicidal behavior: associations with preadolescent physical abuse and selected risk and protective factors. *J Am Acad Child Adolesc Psychiatry.* (2007) 46:859–66. 10.1097/chi.0b013e318054e702 17581450

[57] Thompson MP Kingree JB Desai S. Gender differences in long-term health consequences of physical abuse of children: data from a nationally representative survey. *Am J Public Health.* (2004) 94:599–604. 10.2105/AJPH.94.4.599 15054012 PMC1448305

[58] Collins A Muñoz-Solomando A. The transition from child and adolescent to adult mental health services with a focus on diagnosis progression. *BJPsych Bull.* (2018) 42:188–92. 10.1192/bjb.2018.39 29925438 PMC6189989

[59] Arnold EM Goldston DB Ruggiero A Reboussin BA Daniel SS Hickman EA. Rates and predictors of rehospitalization among formerly hospitalized adolescents. *Psychiatr Serv.* (2003) 54:994–8. 10.1176/appi.ps.54.7.994 12851436

[60] Guillaume S Jaussent I Maimoun L Ryst A Seneque M Villain L Associations between adverse childhood experiences and clinical characteristics of eating disorders. *Sci Rep.* (2016) 6:35761. 10.1038/srep35761 27804994 PMC5090200

[61] Rienecke RD Johnson C Le Grange D Manwaring J Mehler PS Duffy A Adverse childhood experiences among adults with eating disorders: comparison to a nationally representative sample and identification of trauma profiles. *J Eat Disord.* (2022) 10:72. 10.1186/s40337-022-00594-x 35596196 PMC9123748

[62] Hoek HW. Incidence, prevalence and mortality of anorexia nervosa and other eating disorders. *Curr Opin Psychiatry.* (2006) 19:389–94. 10.1097/01.yco.0000228759.95237.78 16721169

[63] Edakubo S Fushimi K. Mortality and risk assessment for anorexia nervosa in acute-care hospitals: a nationwide administrative database analysis. *BMC Psychiatry.* (2020) 20:19. 10.1186/s12888-020-2433-8 31931765 PMC6958629

[64] Wang YR Sun JW Lin PZ Zhang HH Mu GX Cao FL. Suicidality among young adults: unique and cumulative roles of 14 different adverse childhood experiences. *Child Abuse Negl.* (2019) 98:104183. 10.1016/j.chiabu.2019.104183 31521907

[65] Dube SR Anda RF Felitti VJ Chapman DP Williamson DF Giles WH. Childhood abuse, household dysfunction, and the risk of attempted suicide throughout the life span: findings from the adverse childhood experiences study. *JAMA.* (2001) 286:3089–96. 10.1001/jama.286.24.3089 11754674

[66] Chartier MJ Walker JR Naimark B. Separate and cumulative effects of adverse childhood experiences in predicting adult health and health care utilization. *Child Abuse Negl.* (2010) 34:454–64. 10.1016/j.chiabu.2009.09.020 20409586

[67] van der Feltz-Cornelis CM Potters EC van Dam A Koorndijk RPM Elfeddali I van Eck van der Sluijs JF. Adverse childhood experiences (ACE) in outpatients with anxiety and depressive disorders and their association with psychiatric and somatic comorbidity and revictimization. cross-sectional observational study. *J Affect Disord.* (2019) 246:458–64. 10.1016/j.jad.2018.12.096 30599369

[68] Paul KI Moser K. Unemployment impairs mental health: meta-analyses. *J Vocat Behav.* (2009) 74:264–82. 10.1016/j.jvb.2009.01.001

[69] McKee-Ryan F Song Z Wanberg CR Kinicki AJ. Psychological and physical well-being during unemployment: a meta-analytic study. *J Appl Psychol.* (2005) 90:53–76. 10.1037/0021-9010.90.1.53 15641890

[70] Arnett JJ. Emerging adulthood: what is it, and what is it good for? *Child Dev Perspect.* (2007) 1:68–73. 10.1111/j.1750-8606.2007.00016.x

[71] Arnett JJ. Emerging adulthood: a theory of development from the late teens through the twenties. *Am Psychol.* (2000) 55:469–80. 10.1037/0003-066X.55.5.469 10842426

[72] Seiffge-Krenke I. «Emerging adulthood»: forschungsbefunde zu objektiven markern, entwicklungsaufgaben und entwicklungsrisiken. *Z Für Psychiatr Psychol Psychother.* (2015) 63:165–73. 10.1024/1661-4747/a000236

[73] Merrick MT Ports KA Ford DC Afifi TO Gershoff ET Grogan-Kaylor A. Unpacking the impact of adverse childhood experiences on adult mental health. *Child Abuse Negl.* (2017) 69:10–9. 10.1016/j.chiabu.2017.03.016 28419887 PMC6007802

[74] Avinger KA Jones RA. Group treatment of sexually abused adolescent girls: a review of outcome studies. *Am J Fam Ther.* (2007) 35:315–26. 10.1080/01926180600969702

[75] Jorm AF Mulder RT. Prevention of mental disorders requires action on adverse childhood experiences. *Aust N Z J Psychiatry.* (2018) 52:316–9. 10.1177/0004867418761581 29506400

